# A study on the use of strain-specific and homologous promoters for heterologous expression in industrial *Saccharomyces cerevisiae* strains

**DOI:** 10.1186/s13568-018-0613-4

**Published:** 2018-05-21

**Authors:** Daniel Pereira de Paiva, Tiago Benoliel Rocha, Marciano Regis Rubini, André Moraes Nicola, Viviane Castelo Branco Reis, Fernando Araripe Gonçalves Torres, Lidia Maria Pepe de Moraes

**Affiliations:** 1 Departamento de Biologia Celular, Instituto de Ciências Biológicas, Universidade de Brasília, Brasília, DF 70910-900 Brazil; 2 Faculdade de Medicina, Universidade de Brasília, Brasília, DF 70910-900 Brazil

**Keywords:** Amylase, Promoter, Industrial yeast, *Saccharomyces cerevisiae*, Gene expression

## Abstract

Polymorphism is well known in S*accharomyces cerevisiae* strains used for different industrial applications, however little is known about its effects on promoter efficiency. In order to test this, five different promoters derived from an industrial and a laboratory (S288c) strain were used to drive the expression of *eGFP* reporter gene in both cells. The *ADH1* promoter (P_*ADH1*_) in particular, which showed more polymorphism among the promoters analyzed, also exhibited the highest differences in intracellular fluorescence production. This was further confirmed by Northern blot analysis. The same behavior was also observed when the gene coding for secreted α-amylase from *Cryptococcus flavus* was placed under the control of either P_*ADH1*_. These results underline the importance of the careful choice of the source of the promoter to be used in industrial yeast strains for heterologous expression.

## Introduction

It has been previously described that *S. cerevisiae* strains present different patterns of gene expression according to environmental stress factors (James et al. 2003; Kvitek et al. 2008). The Brazilian fermentation process is an adaptation of the Melle-Boinot process where cells are intensively recycled through a process of centrifugation and washing in diluted sulfuric acid resulting in high cell densities (Babrzadeh et al. 2012; Basso et al. 2008; Wheals et al. 1999). This process occurs in non-sterile conditions making it susceptible to contamination and genetically and physiologically adapted strains tend to dominate (da Silva-Filho et al. 2005a; Zaldivar et al. 2002; Zheng et al. 2013). However, the choice of a yeast strain more adapted to the fermentation process is not the only concern that should be considered. The majority of metabolic pathways studies focuses, for example, on the choice of promoters for heterologous expression cassette construction via the selection of appropriate promoters for its strength under different growth conditions (Partow et al. 2010; Peng et al. 2015; Sun et al. 2012), in improving the strength through construction of hybrid promoters (Blazeck et al. 2012) and in optimizing metabolic pathway using combinatorial metabolic libraries (Carquet et al. 2015; Du et al. 2012). But no study to date takes into account the particular genetics of the host strain that will receive the promoters.

In order to determine if commonly used promoters in synthetic biology show different expression patterns, we analyzed and compared five promoters (P_*CYC1*_, P_*TEF1*_, P_*PGK1*_, P_*PGI1*_ and P_*ADH1*_) selected from *S. cerevisiae* strains JPU (industrial) and S288C (laboratory), for the heterologous expression of the *eGFP* gene and the α-amylase gene (*AMY1*) from *C. flavus* (Galdino et al. 2008b).

## Methods

### Strains and media

*Escherichia coli* strain XL-10 Gold {*endA1 glnV44 recA1 thi*-*1 gyrA96 relA1 lac Hte Δ(mcrA)183 Δ(mcrCB*-*hsdSMR*-*mrr)173 tet*^*R*^ *F’[proAB lacI*^*q*^*ZΔM15 Tn10(Tet*^*R*^ *Amy Cm*^*R*^*)]*} was used as host for routine recombinant DNA manipulations. *E. coli* was grown in LB media (1% peptone, 0.5% yeast extract, 1% NaCl, pH 7.2) at 37 °C supplemented with 100 μg/mL ampicillin when necessary. For solid medium, 1.5% agar was added.

*Saccharomyces cerevisiae* strain JPU (*a/α ura3Δ*) (Reis et al. 2012) and S288C (*α SUC2 gal2 mal2 mel flo1 flo8*-*1 hap1 ho bio1 bio6*) were used as a source of genomic DNA. *S. cerevisiae* JPU and CEN.PK2 (*a/a*, *ura3*-*52*, *leu2*-*3112*, *trp1*-*289*, *his3*-*1*) were used as host strains to express eGFP and α-amylase from *Cryptococcus flavus* (Galdino et al. 2008b). JPU is a *ura3* mutant strain derived from *S. cerevisiae* JP1, an industrial strain isolated from Japungu Agroindustrial distillery (Santa Rita-PB, Brazil) and deposited at the Department of Mycology Culture Collection (Universidade Federal de Pernambuco, Brazil).

*Saccharomyces cerevisiae* strains were grown in YPD medium (2% peptone, 1% yeast extract, 2% glucose) or minimum dextrose (MD) medium (0.17% yeast nitrogen base without amino acids, 0.5% ammonium sulfate, 2% glucose) supplemented with 20 mg L^−1^ histidine, 30 mg L^−1^ leucine, 20 mg L^−1^ tryptophan (for CEN.PK2 strain) or 20 mg L^−1^ uracil (for both yeasts) when necessary. For solid medium, 2% agar was added. For enzyme activity assays, YPD medium was supplemented with 1% starch and MD medium was supplemented with 1% starch and Asp-Glu buffer (0.4% l-aspartate, 0.4% l-glutamate, pH 5.5).

### Yeast transformation

Yeast transformation was carried out as previously described (Chen et al. 1992). Yeast cells were spun down from a stationary phase culture into 1.5 mL tubes, vortexed with ONE-STEP buffer [200 mM lithium acetate, 40% polyethylene glycol 3350, 100 mM dithiothreitol (DTT)], 0.2–1 μg of plasmid DNA and 50 μg of single-stranded carrier DNA in a total volume of 100 μL. Cells were incubated at 45 °C for 1 h, washed with sterile deionized water for PEG removal, resuspended and plated directly onto selective medium. Cells were incubated at 28 °C for 2–3 days for isolated colonies.

### DNA manipulation

The yeast genomic DNA extraction used for the PCR reactions was performed as previously described (Drumonde-neves et al. 2013), with modifications. A yeast colony was inoculated into 1 mL of YPD and incubated at 30 °C for 16 h. Briefly, cells were centrifuged at 10,000×*g* for 2 min and the supernatant was discarded. The cell pellet was resuspended in 100 μL of Buffer I (1 M sorbitol, 100 mM EDTA, pH 7.5) containing 26 U lyticase (Sigma-Aldrich) and the cell suspension was incubated at 37 °C for 30 min. A 100 μL solution of Buffer II (50 mM Tris–HCl, 20 mM EDTA, 0.35 M SDS, pH 7.4) was added and the cell suspension was incubated at 65 °C for 5 min and then 80 μL of 5 M potassium acetate was added following incubation at − 20 °C for 5–10 min. The suspension was centrifuged at 10,000×*g* at 4 °C for 15 min and the supernatant was transferred to a new 1.5 mL tube and 250 μL of isopropanol was added following incubation at room temperature for 5 min. The solution was centrifuged at 10,000×*g* for 2 min, the supernatant was removed by aspiration and the pellet was washed with 500 μL of 70% (v/v) ethanol. The pellet was resuspended in 500 μL deionized H_2_O containing 100 μg/mL RNAse A.

Standard cloning, plasmid isolation and bacterial transformations were performed as previously described (Sambrook and Russel 2001). For promoter and probe amplification, the oligonucleotides listed in Table 1 were used. Phusion High-Fidelity DNA Polymerase (Thermo Scientific, Ipswich, MA, USA) or Taq DNA Polymerase (Thermo Fisher Scientific) were used for promoter and probes amplification by PCR, respectively, following the supplier instructions; primers were purchased from Integrated DNA Technologies (Coralvillle, IA, USA) and Exxtend (Campinas, SP, BRA). T4 DNA ligase was purchased from Promega and all restriction enzymes were purchased from New England Biolabs. DNA purification was performed using Wizard SV Gel and PCR Clean-Up System (Promega).

**Table 1:** Primers used in this study

Primers	Fragment (approximate length)	Restriction endonuclease	Sequence (5′→3′)^a^
pPGKSACI-F	PGK1 (1500 bp)	*Sac*I	ttcGAGCTCtctaactgatctatccaaaactga
pPGKBAM-R		*Bam*HI	tGGATCCtgttttatatttgttgtaaaaagtaga
ADHSAC-F	ADH1 (1500 bp)	*Sac*I	aGAGCTCattaaaacaagaagagggttgac
ADHBAM-R		*Bam*HI	aGGATCCtgtatatgagatagttgattgtatg
PGISACI-F	PGI1 (400 bp)	*Sac*I	aGAGCTCgtgggtgtattggattatagg
PGIBAM-R		*Bam*HI	aGGATCCtttttaggctggatcttgattcta
CYCpp5	CYC1 (300 bp)	*Sac*I	tGAGCTCgagctcatttggcgagcgtt
CYCpp3		*Bam*HI	tGGATCCgattagtgtgtgtatttgtgtttg
TEFP5	TEF1 (400 bp)	*Sac*I	gGAGCTCccccacacaccatagcttcaa
TEFP3		*Bam*HI	cGGATCCtttgtaattaaaacttagattagattg
eGFPBAM-F	*eGFP* (770 bp)	*Bam*HI	aGGATCCatggtgagcaagggcgagga
eGFPNOT-R		*Not*I	gGCGGCCGCttacttgtagagctc
GPD5	*ZWF*1 (1500 bp)	*Bam*HI	cGAATTCaagatgagtgaaggccccgt
GPD3		*Bam*HI	cGTCGACattatccttcgtatcttctggc

^a^Restriction site are in capital letters

### Plasmids construction

All plasmids used in this study were based on vector YEp352 vector (Hill et al. 1986), which contains the *URA3* auxotrophic marker. The PGK1 cassette from the YIPGK1 vector (YEp351 based plasmid) was isolated after a double digestion with *Sac*I and *Hin*dIII following ligation into YEp352 linearized with the same restriction enzymes, yielding plasmid Y2PGK1. Then, Y2PGK1 was double digested with *Bam*HI and *Not*I and ligated to the *eGFP* gene from plasmid pEGFP-N3 (Clontech Laboratories), which had been digested with the same enzymes, yielding the vector Y2PGKGFP. In order to obtain the *eGFP* gene under control of different promoters, plasmid Y2PGKGFP was double digested with *Sac*I and *Bam*HI to remove the *PGK1* promoter which was replaced with either P_*ADH1*_, P_*PGK1*_, P_*CYC1*_, P_*TEF1*_ or P_*PGI1*_ amplified from either the *S. cerevisiae* JPU or S288C strains, which had been previously digested with the same enzymes prior to ligation. Plasmids containing promoters from the JPU or from the S288C strains received the prefix Y2J and Y2S, respectively. For the construction of plasmids containing the α-amylase gene from *C. flavus*, plasmid Y2PGK-1 was digested with *Sac*I and *Bam*HI restriction enzymes for removal of P_*PGK1*_, which was replaced with P_*ADH1*_ and P_*PGK1*_ from the *S. cerevisiae* JPU and S288C strains, respectivelly. The *AMY1* gene was obtained from YEpAMY1 (Galdino et al. 2008b), digested with *Bgl*II and ligated into the vectors previously linearized with *Bam*HI. The correct gene orientation was confirmed with digestion with *Bam*HI. The resulting plasmids were denominated Y2JADH-AMY1, Y2SADH-AMY1, Y2JPGK-AMY1 and Y2SPGK-AMY1.

### DNA sequence and analysis of promoters

Plasmids were isolated using the Wizard SV Plus Miniprep DNA Purification System (Promega) and promoters were sequenced by Myleus Biotecnologia (http://www.myleus.com) and Laboratório de Ciências Genômicas e Biotecnologia Molecular (Universidade Católica, Brasília, DF, BRA) using oligonucleotides M13-F (5′-GCTCGTATGTTGTGTGTGTGGAATTG-3′) and eGFP-R2 (5′-GTCCAGCTCGACCAGGAT-3′). The ADH-IR (5′- GCCGCAAAGCCAAATACATCA-3′) oligonucleotide was also used with M13-F for P_*ADH1*_ sequencing and contig assembly. Sequence alignments were performed using CLUSTAL W (Thompson et al. 1994). Reference DNA sequence from *S. cerevisiae* S288C was obtained from the National Center for Biotechnology Information (http://www.ncbi.nlm.nih.gov/).

### Flow cytometry

Single *S. cerevisiae* colonies transformed with different plasmids were picked directly from a Petri dish (in biological triplicate) and inoculated in 500 μL MD medium supplemented with 20 mg L^−1^ histidine, 30 mg L^−1^ leucine, 20 mg L^−1^ tryptophan (for CEN.PK2 strain) or 20 mg L^−1^ uracil (for both control strains), in a deep well plate and incubated in an orbital shaker for 24 h at 28 °C under agitation of 300 rpm. Samples were normalized to an OD_600_ of 0.01 in 500 μL of MD. Reinoculated strains were incubated for another 16 h at 28 °C (300 rpm). About 400 μL of cultures were harvested and centrifuged at 3000×*g* at 4 °C for 5 min, resuspended in the same volume of PBS (8 g L^−1^ NaCl, 0.2 g L^−1^ KCl, 1.44 g L^−1^ Na_2_HPO_4_, 0.24 g L^−1^ KH_2_PO_4_, pH 7.4), normalized to a concentration of no more than 10^6^ cells/mL and analyzed by flow citometer on BD FACSVerse™ (Becton, Dickinson and Company). About 10,000 events were captured, and individual cells were separated from debris and cell clumps by forward scatter (FSC) versus side scatter (SSC) and FL-W versus FL-A plots. Data were analyzed using FlowJo software to compute mean fluorescence values. Day-to-day variability was mitigated by analyzing all comparable strains on the same day. The average fluorescence and standard error were calculated from the mean values of biological replicates, and the statistical analyses were performed with GraphPad Prism 6 (GraphPad Software, Inc.) using Two-way ANOVA (*p* ≤ 0.05).

### RNA extraction

Yeast cells were grown in MD medium for 24 h at 30 °C under 200 rpm agitation and inoculated in 250 mL MD medium with initial OD_600_ of 0.25 following grown until an OD_600_ ~ 0.8. Cultures were treated with 50 µg/mL cycloheximide for 5 min at 30 °C under 200 rpm agitation. Isolation of total RNA was performed with TRIzol^®^ Reagent (Invitrogen), according to manufacturer’s instructions. All RNA samples were stored at − 80 °C.

### Northern blotting

Separation under denaturation conditions and transfer of RNA samples to nylon membranes were performed as previously described (Sambrook and Russel 2001). The ZWF1 probe was amplified from yeast genomic DNA using GPD5 and GPD3 primers and the eGFP probe was amplified from pEGFP-N3 plasmid using eGFPBAM-F and eGFPNOT-R primers. Labelling and detection of DNA probes were made with Amersham Gene Images AlkPhos Direct Labelling and Detection System kit (GE Healthcare Life Sciences, Little Chalfont, Buckinghamshire, GBR), according to manufacturer’s instructions. Images were edited with Adobe Photoshop CS6 (Adobe Systems Incorporated). Densitometry analysis were made with Image Quant TL v. 8.1 (GE Healthcare Life Sciences).

### Enzyme activity assay

Biological samples were prepared in independent triplicates and amylase activity measurements of these samples were performed in technical triplicates for each plasmid. Pre-inoculum of yeasts carrying Y2JADH-AMY1, Y2SADH-AMY1, Y2JPGK-AMY1 and Y2SPGK-AMY1 plasmids were grown in MD medium for 24 h, 30 °C, 300 rpm agitation, and inoculum were made in 50 mL MD medium with initial OD_600_ of 0.25 and grown under the same conditions. Samples were collected at different times for cell growth and enzyme activity measurements.

Enzyme activity was determined by adding 100 μL of 0.5% starch solution to 60 μL of culture supernatant and 40 μL of 0.5 M sodium acetate buffer (pH 6). The reaction was incubated at 40 °C for 10–40 min. The reaction was stopped by adding 200 μL of 1 M acetic acid solution and 200 μL of iodine reagent (0.2% I_2_, 2% KI). The volume of the reaction was completed to 5 mL with distilled water and the absorbance determined at 660 nm. The amylolytic activity was monitored by hydrolysis of starch as described by de Moraes et al. (1995). One unit of amylase activity was defined as the amount of enzyme necessary to hydrolyze 0.1 mg of starch per minute at 40 °C.

## Results

### Cloning of promoters

In order to compare transcription in different genetic backgrounds a set of five promoters (Table 2) commonly used in *S. cerevisiae* were selected and amplified from genomic DNA from JPU (industrial strain) and S288C (laboratory strain). The resulting 10 promoter amplicons were cloned into an expression cassette which includes the *eGFP* reporter gene and the *PGK1* terminator, previously assembled in the multicopy vector YEp352 (Hill et al. 1986). In total, 10 plasmids were constructed. The five plasmids with promoters from JPU strain were named Y2JCYC, Y2JTEF, Y2JPGI, Y2JPGK and Y2JADH, and the five plasmids with promoters from S288C strain were named Y2SCYC, Y2STEF, Y2SPGI, Y2SPGK and Y2SADH. As *S. cerevisiae* JPU is diploid and S288C is haploid, the latter was replaced by the CEN.PK2 a diploid laboratory strain. All ten plasmids were transformed into both yeast hosts, resulting in 20 strains.

**Table 2:** Promoters used in plasmids constructions

Promoter	Name	Accession numbers (GenBank)	
		S288C	JPU
CYC1	CYtochrome C	KY704470	KY704471
TEF1	Translation Elongation Factor	KY704476	KY704477
PGI1	PhosphoGlucoIsomerase	KY704472	KY704473
PGK1	3-PhosphoGlycerate Kinase	KY704474	KY704475
ADH1	Alcohol DeHydrogenase	KY704468	KY704469

### Promoter sequence analysis

Promoters amplified from both JPU and S288C strains were subject to sequencing and compared to the *S. cerevisiae* S288C reference sequences (Goffeau et al. 1996). As expected, no differences in sequence were found in promoters amplified from S288C strain and the sequences deposited in the NCBI and SGD database (Table 2). On the other hand, all promoter sequences derived from JPU strain had at least one altered nucleotide, with the exception of P_*ADH1*_, which featured six nucleotide differences and two deletions (Table 3).

**Table 3:** Nucleotide differences of the promoters from JPU strain

Promoter	Sequence variation
CYC1	− 52C>T
TEF1	− 113C>T; − 308T>A
PGI1	− 229T>C
PGK1	− 91G>T
ADH1	− 1448A>G; − 1432G>A; − 1391G>A; − 1308A>G; − 1173A>C; − 914A>G; − 539A>T; − 387_− 386delCT

### Promoter strength analysis

**Fig. 1:**
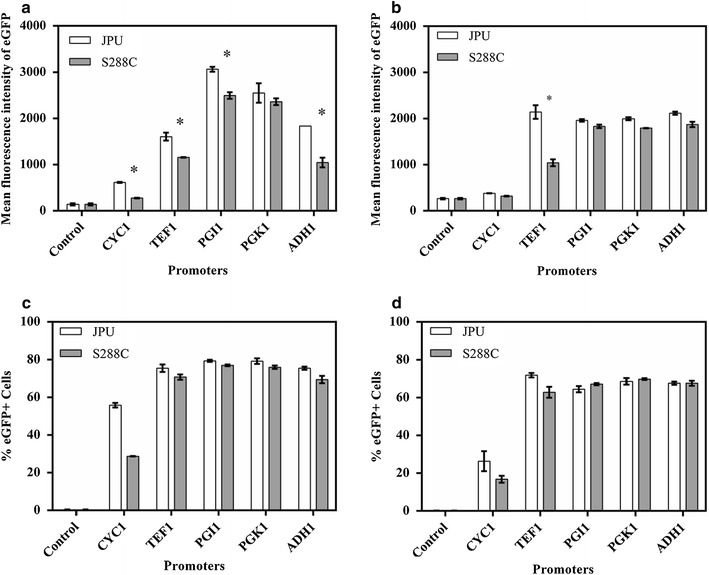
Comparison of promoter strength in different *S. cerevisiae* hosts. Promoters from industrial strain JPU (*white bars*) and laboratory strain S288C (*gray bars*) driving the expression of *eGFP* in JPU (**a**) and CEN.PK2 (**b**) were analyzed by flow cytometry. Only EGFP positive JPU (**c**) and CEN.PK2 (**d**) cells were analyzed for intensity of intracellular fluorescence of EGFP in both cell strains (**c**, **d**). The asterisk symbol indicates promoters with significant difference (Two-way ANOVA, *p* ≤ 0.05) in mean fluorescence. The error bars represent the standard error of biological triplicates

The strength of equivalent promoters from different origins was assessed by analyzing intracellular fluorescence intensity derived from EGFP (Fig. 1). For *S. cerevisiae* JPU industrial strain, the intracellular fluorescence intensity of EGFP showed no significant difference when the reporter gene was under the control of P_*PGK1*_ from either JPU or S288C strains. In contrast, when the reporter gene was placed under the control of P_*CYC1*_, P_*TEF1*_, P_*PGI1*_ and P_*ADH1*_, intracellular fluorescence showed significant differences (Fig. 1a). When the same promoters were analyzed in *S. cerevisiae* CEN.PK2 laboratory strain, intracellular fluorescence intensity showed no significant difference when the reporter gene was under control of P_*CYC1*_, P_*PGK1*_, P_*PGI1*_ and P_*ADH1*_ derived from both strains (Fig. 1b). However, among the promoters that presented significant differences it was observed that P_*TEF1*_ from the industrial strain was stronger than its equivalent from the laboratory strain in both yeast hosts (Fig. 1b). We also noted that in both cell strains the JPU-derived promoters showed greater strength than the S288C-derived promoters. Even in strains that showed no significant difference, the promoters from JPU were slightly stronger than the S288C promoters (Fig. 1).

### Promoter expression

To determine if the differences observed in the intensity of intracellular fluorescence of EGFP were at the transcription level, two promoters were selected for Northern blot analysis. The *ADH1* promoter was selected because it showed the most significant difference in the intracellular fluorescence of EGFP and the greatest number of differences between the JPU and S288C promoter sequences. The *PGK1* promoter was also selected for this analysis because it did not show significant differences neither in sequence or activity in both strains.

**Fig. 2:**
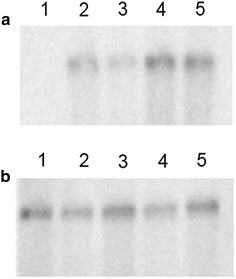
Transcription analysis of *eGFP* gene under control of P_*ADH1*_ and P_*PGK1*_ from JPU and S288C in *S. cerevisiae* JPU. Total yeast RNA was prepared, run in a 1.5% agarose/formaldehyde gel and transferred to a nitrocellulose membrane. The membrane was probed with the *eGFP* PCR product (**a**) and with the *ZWF1* PCR product as loading control (**b**). Lanes: *1* non-transformed cell; *2* Y2JADH; *3* Y2SADH; *4* Y2JPGK; *5* Y2SPGK. The predicted size of *eGFP* mRNA is approximately 0.7 kb and SWF1 mRNA is ~ 1.5 kb

Each transformant was grown to mid-exponential phase in MD media before harvesting and RNA extraction. The *eGFP* PCR product was used as probe in the Northern blot analysis (Fig. 2). Detection with the *eGFP* probe showed differences between mRNA levels when the *eGFP* gene was under the control of P_*ADH1*_ from JPU and S288C, and no significant difference was observed when the same gene was under the control of P_*PGK1*_ in both strains (Fig. 2a). The *SWF1* (Glucose-6-phosphate dehydrogenase) PCR product was used as loading control probe (Fig. 2b). Densitometric analysis showed, after normalization, that the mRNA levels of the *eGFP* gene from Y2JADH were 2.5-fold higher than the levels obtained from Y2SADH, and the mRNA levels of the *eGFP* gene obtained by Y2JPGK and Y2SPGK showed no significant difference.

### Amylase expression under different promoters

**Fig. 3:**
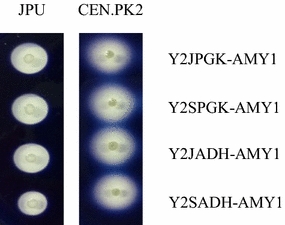
Amylolytic activity of yeast transformants in plate assays. *S. cerevisiae* JPU and CEN.PK2 were transformed with the indicated plasmids and plated in MD medium supplemented with 1% starch. After 24 h (JPU) and 48 h (CEN.PK2) of growth at 30 °C, the plates were stained with iodine vapor. The different growth time between strains was due to the slower growth rate of CEN.PK2 laboratory strain

We sought to assess whether the observed differences in the intensity of intracellular fluorescence of EGFP were also observed for a different gene under the control of P_*ADH1*_ and P_*PGK1*_. For this, the gene coding for EGFP from Y2JADH, Y2SADH, Y2JPGK and Y2SPGK was replaced by the gene encoding the α-amylase from the yeast *C. flavus* (Galdino et al. 2011). The use of an amylase from yeast was aimed at reducing variations that could affect the promoter analysis. These plasmids were transformed into JPU and CEN.PK2 yeast cells which were grown in MD medium. In a qualitative analysis, JPU transformed with Y2JADH-AMY1 and Y2SADH-AMY1 showed different hydrolysis halos sizes while both transformants containing Y2JPGK-AMY1 and transformants containing Y2SPGK-AMY1 showed no significant differences (Fig. 3). This difference in activity was not observed in CEN.PK2 strain, where no significant difference in starch hydrolysis halos between transformants bearing plasmids Y2JADH-AMY1 and Y2SADH-AMY1 or between transformants transformed with Y2JPGK-AMY1 and Y2SPGK-AMY1 was observed (Fig. 3).

**Fig. 4:**
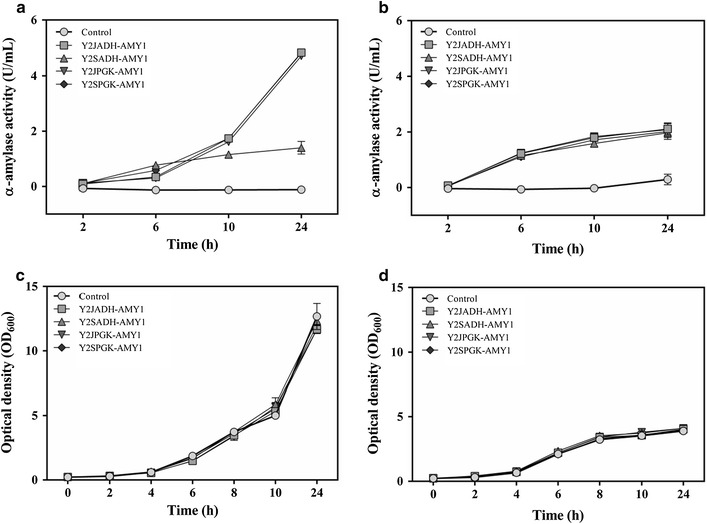
Kinetics of α-amylase from *C. flavus* production by recombinant *S. cerevisiae* JPU and CEN.PK2 host strains. Amylase activity in JPU (**a**) and CEN.PK2 (**b**) strains, and growth curves of JPU (**c**) and CEN.PK2 (**d**) strains. The symbols represent: non-transformed cell (*circle*); Y2JADH-AMY1 (*square*); Y2SADH-AMY1 (*triangle*); Y2JPGK-AMY1 (*inverted triangle*); Y2SPGK-AMY1 (*diamond*). The error bars represent the standard error of biological and experimental triplicates

In a quantitative analysis, the time course of growth and secreted amylase production by JPU and CEN.PK2 transformants during batch culture were evaluated and are shown in Fig. 4. For JPU strain, transformants containing Y2JADH-AMY1 showed a higher activity of α-amylase (4.84 U mL^−1^) than those bearing Y2SADH-AMY1 (1.40 U mL^−1^) after 24 h of cultivation, while cells transformed with Y2JPGK-AMY1 (4.72  U mL^−1^) and Y2SPGK-AMY1 (4.81 U mL^−1^) showed no difference (Fig. 4a). This difference in mean activity was not observed in CEN.PK2 strain, where no significant difference in α-amylase activity between transformants containing plasmids Y2JADH-AMY1 (2.10 U mL^−1^) and Y2SADH-AMY1 (1.96 U mL^−1^) or between transformants containing plasmids Y2JPGK-AMY1 (2.08 U mL^−1^) and Y2SPGK-AMY1 (2.01  U mL^−1^) was observed (Fig. 4b). Analysis of the cell growth curve showed no difference in cell growth of the analyzed clones, indicating that the difference in α-amylase extracellular activity was due to the difference in α-amylase production under the control of these promoters and not due to a difference in the number of cells in the culture medium (Fig. 4c, d).

After normalization of the enzymatic activity by the OD of the cells (data not shown), activity values were similar in both strains for all promoters except for P_*ADH1*_ from S288C strain used in the JPU host, which showed lower enzymatic activity, indicating that this promoter did not work properly in this strain (Fig. 4). It was also possible to observe that the highest enzymatic activity detected in the JPU strain was due to its higher cell growth.

## Discussion

*Saccharomyces cerevisiae* is the most important micro-organism employed in the production of bioethanol. This is due to several characteristics such as high ethanol productivity, tolerance to production stresses and resistance to fermentation by-products (Zaldivar et al. 2002; Zheng et al. 2013). A study of the resistance to different types of stress carried out in yeast strains used for wine production (Ivorra et al. 1999) was used as a basis for understanding the physiology of ethanol-producing strains isolated from Brazilian sugarcane mills. The study conducted by (Della-Bianca and Gombert 2013) compared the physiology of the main strains used in the production of fuel ethanol in Brazil with some well-known laboratory strains, evaluating their tolerance in relation to the classic stress factors and the specific ones faced in the industrial process of fermentation.

Due to the specifics characteristics of ethanol production in Brazil, the manipulation of laboratory yeasts, as well as of strains used in other fermentation processes, and their subsequent adaptation to the industrial use is not a viable option, since this strategy is based on the use of strains that are unable to endure the stressful environment of the fermentation vats, being quickly replaced by more adapted wild strains (Abreu-Cavalheiro and Monteiro 2013; Basso et al. 1993). *S. cerevisiae* JP1 (and its derivative JPU) demonstrated to be dominant in fermentation vats and to possess desirable characteristics in strains used in the production of ethanol (Della-Bianca and Gombert 2013; da Silva-Filho et al. 2005a, b), making this strain a suitable candidate for genetic modifications and use in the industrial process.

Besides the choice of the micro-organism suitable for the process, selection of suitable promoters for heterologous expression is an important step in metabolic pathways engineering. The majority of metabolic pathways studies focuses, for example, on the choice of promoters for heterologous expression cassette construction via the selection of appropriate promoters for its strength under different growth conditions (Partow et al. 2010; Peng et al. 2015; Sun et al. 2012), in improving the strength through construction of hybrid promoters (Blazeck et al. 2012) and in optimizing metabolic pathway using combinatorial metabolic libraries (Carquet et al. 2015; Du et al. 2012). But no study to date, takes into account in determining the promoter strength considering its genome of origin and the host strain that will receive them.

In this work we tested five promoters commonly used for heterologous expression in *S. cerevisiae* were tested in different genetic backgrounds. Sequence analysis showed most promoters derived form an industrial strain showed only one nucleotide variation, with the exception of the P_*ADH1*_ which featured the highest number of differences, including six nucleotide changes and deletions. Since all the promoters from the JPU strain had at least one nucleotide change, we believe that the differences in intracellular fluorescence of EGFP may have occurred due to changes in regulation of transcription initiation. The industrial strains present a genome of more complex constitution with variable number of chromosomes and ploidy that can contribute to adaptation to the process of fermentation in industrial scale (Carreto et al. 2008; Lucena et al. 2007). In addition, genomes of the industrial strains also present nucleotide variation, due to single nucleotide polymorphisms (SNPs), insertions and deletions (In-Dels), new open reading frames (ORFs), copy number variation (CNV) at the ends of the chromosomes and differences in transcription under stress-fermentative conditions (Akao et al. 2011; Argueso et al. 2009; Borneman et al. 2011; Dunn et al. 2012; Kvitek et al. 2008; Zheng et al. 2013).

The strength of promoters was first assessed by measuring the intracellular fluorescence of EGFP in JPU and CEN.PK2 strains of *S. cerevisiae*. The results obtained with fluorescence analysis demonstrated that P_*CYC1*_, P_*TEF1*_, P_*PGI1*_ and P_*ADH1*_ when tested in the JPU strain showed significantly differences in expression levels (Fig. 1a). In the laboratory CEN.PK2 strain, no significant difference in expression levels was observed, with the exception of P_*TEF1*_ that showed similar results to those obtained in JPU (Fig. 1b). Despite providing useful information, measuring the forces of the promoters is a complicated analysis because there is no consensus since the results vary according to the microorganism used and the culture conditions (Partow et al. 2010; Peng et al. 2015; Sun et al. 2012); and this type of analysis previously performed on heterologous expression studies is an important step.

Promoters P_*PGK1*_ and P_*ADH1*_ were further analyzed by placing the *AMY1* gene from *C. flavus*, under control of promoters from JPU and S288C strains. This α-amylase has been selected because it is of eukaryotic origin and has been shown to function properly in *S. cerevisiae* (Galdino 2008; Galdino et al. 2008a). Thus, we try to eliminate the incompatibility variable of the gene to be expressed with the host yeast (Brat et al. 2009; Hoshida et al. 2013; Ilmén et al. 2011).

With the intracellular analysis performed, the *ADH1* and *PGK1* promoters were selected because they presented the greatest difference in the intracellular expression of EGFP (and in their nucleotide sequence) and did not present significant difference, respectively (Fig. 1a). When analyzing the extracellular activity of α-amylase, the same pattern of the intracellular fluorescence intensity of EGFP was observed both in the formation of starch hydrolysis halos (Fig. 3) and in the enzymatic activity (Fig. 4). Taken together, these results indicate that the difference in strength of these promoters could be occurring at the transcriptional level. The transcripts analysis by Southern blotting obtained when *eGFP* was under control of the *ADH1* promoters indicated that there is a difference in gene transcription. The same was not observed in the *eGFP* transcripts under control of the *PGK1* promoters (Fig. 2).

An earlier study comparing laboratory and wine-producing yeasts has shown that differences in the promoter region alter the expression profile of the same gene in different strains (Hauser et al. 2001). However, the study did not verify whether the same promoters of different origins would behave in the same way in the same host strain. In this work, promoters of different origins were tested in the same yeast hosts. previous studies have shown that different strains of yeasts present different expression patterns when subjected to the same stress factors (Kvitek et al. 2008; Tirosh et al. 2006; Zheng et al. 2013), even those that are used in similar industrial processes such as the production of ethanol, wine and sake (Kvitek et al. 2008; Zheng et al. 2011, 2013).

Despite the differences observed in intracellular fluorescence intensity of eGFP and mRNA expression levels, activity of α-amylase expressed by Y2JADH-AMY1, Y2JPGK-AMY1 and Y2SPGK-AMY1 were similar when expressed in JPU (Fig. 3a). We speculate that the probable cause of such similarity in amylase activity produced by these three plasmids might be attributable to the requirement for enzyme secretion to the extracellular space. The probable cause of such α-amylase activity is the enzyme secretion to the extracellular space, since the secretory production of heterologous enzymes need to be transcribed, translated, folded and secreted. Multicopy vectors were used for overexpression of proteins under the selected promoters, thus leading to an overload in the yeast secretory system and the subsequent limit of extracellular enzyme activity (Bae et al. 2016; Hou et al. 2012; Mattanovich et al. 2004; Wittrup and Robinson 1995).

Genes with specific structural or functional characteristics show higher levels of expression variation across strains, either in response to selection (Tirosh et al. 2006) or mutation accumulation (Landry et al. 2007). It is plausible to speculate that the expression differences between the promoters of strains S288C and JP1, when expressed in JPU, may be related to a combination of factors. Among the promoters analyzed, p*ADH1* from JPU presented more differences in its nucleotide sequence. These nucleotide variations may not be the only factor influencing the difference in expression, but possibly it has an important role (Hauser et al. 2001). It has been shown that the *ADH1* expression increases when *S. cerevisiae* grows under anaerobic conditions (Van Den Brink 2008), the intracellular accumulation of pyruvate directs this metabolite to the alcoholic fermentation pathway (Van Dijken et al. 1993) and the high flow of glycolytic pathway is followed by fermentation even in aerobic conditions (Crabtree effect) (Pronk et al. 1996). The low aeration and high concentrations of sugar conditions in which this yeast was isolated from, may have directed its adaptation to a higher flow of glycolytic pathway and consequent intracellular pyruvate accumulation, which directed to the fermentative pathway that must work quickly to maintain redox balance (Bruinenberg et al. 1983; van Maris et al. 2006). Further studies should be performed to verify this hypothesis.

On account of being a peculiar process, Brazilian ethanol production offers a hostile environment for yeasts where only the most adapted are successful. The manipulation of laboratory strains for later adaptation to the fermentation vats is not a recommended strategy since the implementation of these strains can be very difficult or even impossible. The recommended would be the modification of strains isolated from the fermentation vats, which are already adapted to the hostile environment (Della-Bianca et al. 2013; Steensels et al. 2014). Another point is the choice of promoters used in the genetic modifications of industrial yeasts. Our knowledge about industrial yeasts is not as vast as the knowledge about laboratory yeasts and it is still necessary to learn a lot about the functioning of these yeasts. We have shown in this study that the strength of promoters varies according to the strain of origin and the host strain to be used. Therefore, we suggest that the promoters to be used in industrial yeasts should be tested before they are used to confirm whether they function properly in the host cell or that preferably the promoters of the manipulated yeast are used to avoid problems in the heterologous expression system.

## References

[CR1] Abreu-Cavalheiro A, Monteiro G (2013). Solving ethanol production problems with genetically modified yeast strains. Braz J Microbiol.

[CR2] Akao T, Yashiro I, Hosoyama A, Kitagaki H, Horikawa H, Watanabe D, Akada R, Ando Y, Harashima S, Inoue T, Inoue Y, Kajiwara S, Kitamoto K, Kitamoto N, Kobayashi O, Kuhara S, Masubuchi T, Mizoguchi H, Nakao Y, Nakazato A, Namise M, Oba T, Ogata T, Ohta A, Sato M, Shibasaki S, Takatsume Y, Tanimoto S, Tsuboi H, Nishimura A, Yoda K, Ishikawa T, Iwashita K, Fujita N, Shimoil H (2011). Whole-genome sequencing of sake yeast *Saccharomyces cerevisiae* Kyokai no. 7. DNA Res.

[CR3] Argueso JL, Carazzolle MF, Mieczkowski PA, Duarte FM, Netto OVC, Missawa SK, Galzerani F, Costa GGL, Vidal RO, Noronha MF, Dominska M, Andrietta MGS, Andrietta SR, Cunha AF, Gomes LH, Tavares FCA, Alcarde AR, Dietrich FS, McCusker JH, Petes TD, Pereira GAG (2009). Genome structure of a *Saccharomyces cerevisiae* strain widely used in bioethanol production. Genome Res.

[CR4] Babrzadeh F, Jalili R, Wang C, Shokralla S, Pierce S, Robinson-Mosher A, Nyren P, Shafer RW, Basso LC, de Amorim HV, de Oliveira AJ, Davis RW, Ronaghi M, Gharizadeh B, Stambuk BU (2012). Whole-genome sequencing of the efficient industrial fuel-ethanol fermentative *Saccharomyces cerevisiae* strain CAT-1. Mol Genet Genomics.

[CR5] Bae JH, Sung BH, Seo JW, Kim CH, Sohn JH (2016). A novel fusion partner for enhanced secretion of recombinant proteins in *Saccharomyces cerevisiae*. Appl Microbiol Biotechnol.

[CR6] Basso LC, Oliveira AJ, de Orelli VF, Campos AA, Gallo CR, de Amorim HV (1993). Dominância das leveduras contaminantes sobre as linhagens industriais avaliada pela técnica da cariotipagem. An do Congr Nac da STAB.

[CR7] Basso LC, de Amorim HV, de Oliveira AJ, Lopes ML (2008). Yeast selection for fuel ethanol production in Brazil. FEMS Yeast Res.

[CR8] Blazeck J, Garg R, Reed B, Alper HS (2012). Controlling promoter strength and regulation in *Saccharomyces cerevisiae* using synthetic hybrid promoters. Biotechnol Bioeng.

[CR9] Borneman AR, Desany BA, Riches D, Affourtit JP, Forgan AH, Pretorius IS, Egholm M, Chambers PJ (2011). Whole-genome comparison reveals novel genetic elements that characterize the genome of industrial strains of *Saccharomyces cerevisiae*. PLoS Genet.

[CR10] Brat D, Boles E, Wiedemann B (2009). Functional expression of a bacterial xylose isomerase in *Saccharomyces cerevisiae*. Appl Environ Microbiol.

[CR11] Bruinenberg PM, de Bot PHM, van Dijken JP, Scheffers WA (1983). The role of redox balances in the anaerobic fermentation of xylose by yeasts. Eur J Appl Microbiol Biotechnol.

[CR12] Carquet M, Pompon D, Truan G (2015). Transcription interference and ORF nature strongly affect promoter strength in a reconstituted metabolic pathway. Front Bioeng Biotechnol.

[CR13] Carreto L, Eiriz MF, Gomes AC, Pereira PM, Schuller D, Santos MAS (2008). Comparative genomics of wild type yeast strains unveils important genome diversity. BMC Genomics.

[CR14] Chen D, Yang B, Kuo T (1992). One-step transformation of yeast in stationary phase. Curr Genet.

[CR15] da Silva-Filho EA, dos Santos SKB, Resende ADM, de Morais JOF, de Morais MA, Simões DA (2005). Yeast population dynamics of industrial fuel-ethanol fermentation process assessed by PCR-fingerprinting. Antonie Van Leeuwenhoek.

[CR16] da Silva-Filho EA, de Melo HF, Antunes DF, dos Santos SKB, do Monte Resende A, Simões DA, de Morais MA (2005). Isolation by genetic and physiological characteristics of a fuel-ethanol fermentative *Saccharomyces cerevisiae* strain with potential for genetic manipulation. J Ind Microbiol Biotechnol.

[CR17] de Moraes LMP, Astolfi-Filho S, Oliver SG (1995). Development of yeast strains for the efficient utilisation of starch: evaluation of constructs that express alpha-amylase and glucoamylase separately or as bifunctional fusion proteins. Appl Environ Microbiol.

[CR18] Della-Bianca BE, Gombert AK (2013). Stress tolerance and growth physiology of yeast strains from the Brazilian fuel ethanol industry. Antonie van Leeuwenhoek Int J Gen Mol Microbiol.

[CR19] Della-Bianca BE, Basso TO, Stambuk BU, Basso LC, Gombert AK (2013). What do we know about the yeast strains from the Brazilian fuel ethanol industry?. Appl Microbiol Biotechnol.

[CR20] Drumonde-neves J, Vieira E, Lima MT, Araujo I, Casal M, Schuller D (2013). An easy, quick and cheap high-throughput method for yeast DNA extraction from microwell plates. J Microbiol Methods.

[CR21] Du J, Yuan Y, Si T, Lian J, Zhao H (2012). Customized optimization of metabolic pathways by combinatorial transcriptional engineering. Nucleic Acids Res.

[CR22] Dunn B, Richter C, Kvitek D (2012). Analysis of the *Saccharomyces cerevisiae* pan-genome reveals a pool of copy number variants distributed in diverse yeast strains from differing industrial environments. Genome Res.

[CR23] Galdino AS. Clonagem e expressão de uma α-amilase de *Criptococcus flavus* e sua aplicação na degradação do amido. Universidade de Brasília; 2008

[CR24] Galdino AS, Ulhoa CJ, Moraes LMP, Prates MV, Bloch C, Torres FAG (2008). Cloning, molecular characterization and heterologous expression of AMY1, an a-amylase gene from *Cryptococcus flavus*. FEMS Microbiol Lett.

[CR25] Galdino AS, Ulhoa CJ, Moraes LMP, Prates MV, Bloch C, Torres FAG (2008). Cloning, molecular characterization and heterologous expression of *AMY1*, an alpha-amylase gene from *Cryptococcus flavus*. FEMS Microbiol Lett.

[CR26] Galdino AS, Silva RN, Lottermann MT, Alvares ACM, de Moraes LMP, Torres FAG, de Freitas SM, Ulhoa CJ (2011). Biochemical and structural characterization of Amy1: an alpha-amylase from *Cryptococcus flavus* expressed in *Saccharomyces cerevisiae*. Enzyme Res.

[CR27] Goffeau A, Barrell B, Bussey H, Davis R (1996). Life with 6000 genes. Science.

[CR28] Hauser NC, Fellenberg K, Gil R, Bastuck S, Hoheisel JD, Pérez-Ortín JE (2001). Whole genome analysis of a wine yeast strain. Comp Funct Genomics.

[CR29] Hill JE, Myers AM, Koerner TJ, Tzagoloff A (1986). Yeast/*E. coli* shuttle vectors with multiple unique restriction sites. Yeast.

[CR30] Hoshida H, Fujita T, Cha-Aim K, Akada R (2013). *N*-glycosylation deficiency enhanced heterologous production of a *Bacillus licheniformis* thermostable α-amylase in *Saccharomyces cerevisiae*. Appl Microbiol Biotechnol.

[CR31] Hou J, Tyo KEJ, Liu Z, Petranovic D, Nielsen J (2012). Metabolic engineering of recombinant protein secretion by *Saccharomyces cerevisiae*. FEMS Yeast Res.

[CR32] Ilmén M, Den Haan R, Brevnova E, McBride J, Wiswall E, Froehlich A, Koivula A, Voutilainen SP, Siika-Aho M, La Grange DC, Thorngren N, Ahlgren S, Mellon M, Deleault K, Rajgarhia V, Van Zyl WH, Penttilä M (2011). High level secretion of cellobiohydrolases by *Saccharomyces cerevisiae*. Biotechnol Biofuels.

[CR33] Ivorra C, Pérez-Ortín JE, Del Olmo ML (1999). An inverse correlation between stress resistance and stuck fermentations in wine yeasts. A molecular study. Biotechnol Bioeng.

[CR34] James TC, Campbell S, Donnelly D, Bond U (2003). Transcription profile of brewery yeast under fermentation conditions. J Appl Microbiol.

[CR35] Kvitek DJ, Will JL, Gasch AP (2008). Variations in stress sensitivity and genomic expression in diverse *S. cerevisiae* isolates. PLoS Genet.

[CR36] Landry CR, Lemos B, Rifkin SA, Dickinson WJ, Hartl DL (2007). Genetic properties influencing the evolvability of gene expression. Science.

[CR37] Lucena BTL, Silva-Filho EA, Coimbra MRM, Morais JOF, Simões DA, Morais MA (2007). Chromosome instability in industrial strains of *Saccharomyces cerevisiae* batch cultivated under laboratory conditions. Genet Mol Res.

[CR38] Mattanovich D, Gasser B, Hohenblum H, Sauer M (2004). Stress in recombinant protein producing yeasts. J Biotechnol.

[CR39] Partow S, Siewers V, Bjørn S, Nielsen J, Maury J (2010). Characterization of different promoters for designing a new expression vector in *Saccharomyces cerevisiae*. Yeast.

[CR40] Peng B, Williams TC, Henry M, Nielsen LK, Vickers CE (2015). Controlling heterologous gene expression in yeast cell factories on different carbon substrates and across the diauxic shift: a comparison of yeast promoter activities. Microb Cell Fact.

[CR41] Pronk J, Steensma H, Van Dijken J (1996). Pyruvate metabolism in *Saccharomyces cerevisiae*. Yeast.

[CR42] Reis VCB, Nicola AM, Neto ODSO, Batista VDF, de Moraes LMP, Torres FAG (2012). Genetic characterization and construction of an auxotrophic strain of *Saccharomyces cerevisiae* JP1, a Brazilian industrial yeast strain for bioethanol production. J Ind Microbiol Biotechnol.

[CR43] Sambrook J, Russel DW (2001). Molecular cloning: a laboratory manual.

[CR44] Steensels J, Snoek T, Meersman E, Nicolino MP, Voordeckers K, Verstrepen KJ (2014). Improving industrial yeast strains: exploiting natural and artificial diversity. FEMS Microbiol Rev.

[CR45] Sun J, Shao Z, Zhao H, Nair N, Wen F, Xu J-H, Zhao H (2012). Cloning and characterization of a panel of constitutive promoters for applications in pathway engineering in *Saccharomyces cerevisiae*. Biotechnol Bioeng.

[CR46] Thompson JD, Higgins DG, Gibson TJ (1994). CLUSTAL W: improving the sensitivity of progressive multiple sequence alignment through sequence weighting, position-specific gap penalties and weight matrix choice. Nucleic Acids Res.

[CR47] Tirosh I, Weinberger A, Carmi M, Barkai N (2006). A genetic signature of interspecies variations in gene expression. Nat Genet.

[CR48] Van Den Brink J (2008). Dynamics of glycolytic regulation during adaptation of *Saccharomyces cerevisiae* to fermentative metabolism. Appl Environ Microbiol.

[CR49] Van Dijken JR, Weusthuis RA, Pronk JT (1993). Kinetics of growth and sugar consumption in yeasts. Antonie Van Leeuwenhoek.

[CR50] van Maris AJA, Abbott DA, Bellissimi E, van den Brink J, Kuyper M, Luttik MAH, Wisselink HW, Scheffers WA, van Dijken JP, Pronk JT (2006). Alcoholic fermentation of carbon sources in biomass hydrolysates by *Saccharomyces cerevisiae*: current status. Antonie van Leeuwenhoek Int J Gen Mol Microbiol.

[CR51] Wheals A, Basso L, Alves D, Amorim H (1999). Fuel ethanol after 25 years. Focus (Madison).

[CR52] Wittrup KD, Robinson AS (1995). Constitutive overexpression of secreted heterologous proteins decreases extractable heavy chain binding protein and protein disulfide isomerase levels in *Saccharomyces cerevisiae*. Biotechnol Prog.

[CR53] Zaldivar J, Borges A, Johansson B, Smits HP, Villas-Bôas SG, Nielsen J, Olsson L (2002). Fermentation performance and intracellular metabolite patterns in laboratory and industrial xylose-fermenting *Saccharomyces cerevisiae*. Appl Environ Microbiol.

[CR54] Zheng DQ, Wu XC, Tao XL, Wang PM, Li P, Chi XQ, Li YD, Yan QF, Zhao YH (2011). Screening and construction of *Saccharomyces cerevisiae* strains with improved multi-tolerance and bioethanol fermentation performance. Bioresour Technol.

[CR55] Zheng DQ, Liu TZ, Chen J, Zhang K, Li O, Zhu L, Zhao YH, Wu XC, Wang PM (2013). Comparative functional genomics to reveal the molecular basis of phenotypic diversities and guide the genetic breeding of industrial yeast strains. Appl Environ Microbiol.

